# Epsin 1 Promotes Synaptic Growth by Enhancing BMP Signal Levels in Motoneuron Nuclei

**DOI:** 10.1371/journal.pone.0065997

**Published:** 2013-06-19

**Authors:** Phillip A. Vanlandingham, Taylor R. Fore, Lerin R. Chastain, Suzanne M. Royer, Hong Bao, Noreen E. Reist, Bing Zhang

**Affiliations:** 1 Department of Biology, University of Oklahoma, Norman, Oklahoma; 2 Department of Biomedical Sciences, Molecular, Cellular, and Integrative Neuroscience Program, Colorado State University, Fort Collins, Colorado; University of Iowa, United States of America

## Abstract

Bone morphogenetic protein (BMP) retrograde signaling is crucial for neuronal development and synaptic plasticity. However, how the BMP effector phospho-Mother against decapentaplegic (pMad) is processed following receptor activation remains poorly understood. Here we show that *Drosophila* Epsin1/Liquid facets (Lqf) positively regulates synaptic growth through post-endocytotic processing of pMad signaling complex. Lqf and the BMP receptor Wishful thinking (Wit) interact genetically and biochemically. *lqf* loss of function (LOF) reduces bouton number whereas overexpression of *lqf* stimulates bouton growth. Lqf-stimulated synaptic overgrowth is suppressed by genetic reduction of *wit*. Further, synaptic pMad fails to accumulate inside the motoneuron nuclei in *lqf* mutants and *lqf* suppresses synaptic overgrowth in *spinster* (*spin*) mutants with enhanced BMP signaling by reducing accumulation of nuclear pMad. Interestingly, *lqf* mutations reduce nuclear pMad levels without causing an apparent blockage of axonal transport itself. Finally, overexpression of Lqf significantly increases the number of multivesicular bodies (MVBs) in the synapse whereas *lqf* LOF reduces MVB formation, indicating that Lqf may function in signaling endosome recycling or maturation. Based on these observations, we propose that Lqf plays a novel endosomal role to ensure efficient retrograde transport of BMP signaling endosomes into motoneuron nuclei.

## Introduction

A striking feature of the nervous system is the high degree of synaptic plasticity that enables the brain to process and store the information it receives [1]. Neuronal plasticity requires highly orchestrated signaling networks to transmit extracellular and intracellular cues locally and to the nucleus. Once signals are produced, proper spatio-temporal transmission of these signals depends largely on the intracellular trafficking machinery that controls progression through the endocytic pathway and vesicle trafficking. Although many of the molecules directly involved in the signal transduction cascades that regulate synaptic plasticity have been elucidated, how the intracellular trafficking machinery is coordinated to regulate these signals remains largely unknown.

The *Drosophila* larval neuromuscular junction (NMJ) is a well-characterized system for the study of cell signaling and synaptic development [2]. Synaptic boutons on body wall muscles form in early embryonic stages and grow rapidly as the muscle size is dramatically expanded during second and third instar stages. The rapid NMJ growth is critical for synaptic homeostasis and depends on a myriad of molecular signaling pathways [3], [4]. Primary among these is Bone morphogenetic protein (BMP) signaling, a highly conserved signal transduction pathway. Activation of BMP signaling is initiated when the ligand, Glass bottom boat (Gbb), is released from the postsynaptic muscle and binds to the tetrameric membrane receptor complex in synaptic termini. In developing *Drosophila* NMJs, the receptor complex consists of two BMP type-I receptors, Saxophone (Sax) and Thickveins (Tkv), in addition to the type-II BMP receptor, Wishful thinking (Wit) [4]–[6]. Upon ligand binding, the constitutively active Type II receptor recruits and phosphorylates the Type I receptor. This, in turn, results in Type I receptor-induced phosphorylation of the downstream effector Mother against decapentaplegic (Mad). Current data suggest that pMad itself is not transported along the axon but rather signaling endosomes containing the dimerized BMP receptors are translocated to the nucleus, where Mad is phosphorylated and functions as a transcription factor [7]–[9]. Disruption of this signaling cascade at the ligand, receptor, or transcription factor level severely hinders synaptic growth at the NMJ [5], [6], [10]–[14] (reviewed by [15]).

Liquid facets (Lqf), the *Drosophila* homolog of Epsin1 [16], has been characterized as an endocytic protein with multiple scaffolding domains that allow for binding to phosphoinositol lipids within the cell membrane, clathrin, the AP2 adapter complex, and other endocytic proteins [17], [18]. Lqf also has multiple ubiquitin-interacting motifs and is a key substrate of the de-ubiquitinating enzyme Fat facets (Faf) [18], [19]. It is now accepted that Lqf does not act as a general endocytotic protein, but rather it selectively regulates the endocytosis of some ligands due to its ability to sort ligands in an ubiquitin-dependent manner [20], [21]. In yeast, Epsin1 is found to play a role in endocytosis as well as regulate the actin cytoskeleton [22], [23]. At the fly NMJ, Lqf does not play a role in clathrin-mediated endocytosis of synaptic vesicles (SVs) but it regulates ubiquitin-dependent synaptic development [24], [25].

It remains unclear the mechanism by which Lqf regulates synaptic development and whether Lqf functions in regulating BMP signaling. Here, we present several lines of evidence that Lqf promotes synaptic growth by maintaining pMad levels inside motoneuron nuclei without detectable blockage of axonal transport itself. Our study reveals that Lqf promotes multivesicular body (MVB) formation and plays a key role in relaying BMP signaling from the synaptic terminal to motoneuronal nuclei.

## Materials and Methods

### Fly Strains

All stocks were maintained on standard cornmeal medium at 25°C. These *Drosophila* stocks were used as follows: Elav^C155^-Gal4 (a gift from C. Goodman); *endo* ^A^ and *endo* ^Δ4^ [26]; *wit* ^B11^/*TM6B*, *wit* ^A12^/*TM6B*, and *Df(3L)C175*/*TM6B* (hereafter referred to as *wit Df*) [6]; *lqf* ^ARI^, *lqf* ^FDD9^, UAS-Lqf, and *Df(3L)pbl-X1*/*TM6B* (Lqf Df) [17], [24]; UAS-*Lqf*::Flag (Bloomington #25104); *spin* ^4^/CyOKrGFP and *spin* ^5^/CyOKrGFP [27], and *nwk* ^1^/TM6B [28], [29]. *wit^A12^, lqf Df/TM6B and wit Df, lqf^ARI^*/*TM6B*; *spin4/CyOKrGFP; lqf Df*/*TM6B* and *spin^5^/CyOKrGFP; lqf^ARI^/TM6B* were generated through mitotic recombination and used to generate *lqf*, *wit* or *spin; lqf* double mutants. All other stocks were obtained from Bloomington *Drosophila* stock center.

### Intensity Quantification and Statistical Analysis

Images for pMad intensity quantification were taken at consistent PMT voltage and laser power based upon an initial reference setting for staining groups. Reference settings for imaging pMad at NMJs and inside motoneuron nuclei of the ventral nerve cord (VNC) were based upon *nwk*, or *spin* mutants. Raw images were quantified using a custom ImageJ macro, available upon request. For NMJ pMad intensity quantification, an ROI mask was created based upon a thresholded 2D projection of the HRP channel, mean grayscale levels was measured on a 2D projection of pMad channel on muscle 4, segments A2–A3. For VNC quantification, ROI were created using the ellipse selection tool on 2D projections of pMad. Figures were prepared using Photoshop, without making adjustments to pixel intensity or color brightness.

Statistical analysis was performed using GraphPad Prism Software. Statistical significance was calculated using one-way analysis of variance (ANOVA) followed by Tukey's posttest. Significance is indicated with asterisks: * = p<0.05; ** = p<0.01; *** = p<0.001.

### Electron Microscopy and Analysis

Third instar NMJ tissues were processed as previously described [30]. Briefly, they were dissected in Ca^2+^- free saline (HL-3 pH 7.2, [31], fixed for 1 h in 1% acrolein, 2.5% glutaraldehyde in 0.1 M sodium cacodylate (Cac) buffer (pH 7.2). After washing, larvae were post-fixed for 1 h in 0.5% OsO4, 0.8% KFeCn in 0.1 M Cac, incubated in 5% uranyl acetate for 1 h to overnight, dehydrated with graded ethanols and embedded in EmBed 812 Araldite. 60–90 nm nanometer sections were cut using a diamond knife and Reichert Ultracut E ultramicrotome and post-stained with uranyl acetate and Reynold's lead citrate. Images were captured using a JEOL JEM 2000 EX-II TEM operated at 100 kV and 12,000× magnification. Negatives were digitized using an AGFA Duoscan T2500 at 1500 dpi.

Analysis of vesicle diameter was based upon three CS samples, three Lqf O/E larvae, and three *lqf* null mutants. Type Ib boutons featuring small clear vesicles [32], [33] on muscle 6 were used for quantification. Raw Images were imported into ImageJ (NIH) at a scale setting of 0.732 pixels/nm, feret diameter and area was determined for vesicles within synaptic boutons. Vesicles, under 50 nm in diameter, were omitted from final analysis of endosomal population. Immature and mature MVBs were determined based upon the presence of a double walled membrane vacuole containing at least one internal vesicle. Figures were prepared from raw images and adjusted for brightness and contrast, scale calibration, using Photoshop (Adobe).

### Co-immunoprecipitation

Adult fly heads were isolated from wild-type Canton S and transgenic flag-tagged Lqf (w; p[w, 2× Flag-Lqf]). 2.5 ml of whole flies were flash frozen in liquid nitrogen, vortexed, and passed through a small sieve (no. 25, 710 µm, Precision Eforming, LLC) to allow for separation of fly heads. 100 µl of heads were homogenized using a pestle in lysis buffer [10 mM Hepes, 0.1 mM MgCl_2_, 150 mM NaCl, 5 mM NEM, 2 mM PMSF, and Protease Inhibitor Cocktail (Roche) and spun at 13,000 rpm for 15 minutes at 4°C. Spin was repeated, 50 µl was taken for input, and the remaining lysate was subjected to immunoprecipitation using one of two alternative methods.

In the first method, cell lysates were pre-cleared with mouse IgG Agarose beads (Sigma) for 2 h at 4°C followed by incubation with anti-flag M2 Affinity Gel (Sigma) over night at 4°C. Anti-Flag beads were spun at 5,000× g for 30 sec and 100 µl of supernatant was removed with a narrow-end pipette. The gel was washed 3 times with 500 µl lysis buffer with protease inhibitors allowing time for beads to settle after each spin. Elution of Flag proteins was performed by competition with a Flag peptide. 100 µl of 3X Flag elution solution (150 µg/ml) was added to resin and incubated for 30 minutes with gentle shaking at 4°C. Centrifuged sample at 8,200× g for 30 seconds, removed supernatant and diluted 20 µl in sample buffer. Input, supernatant, and immunoprecipitate samples were then subjected to SDS-Page electrophoresis (10% gel) and blotted with guinea pig anti-Lqf (1∶1000) [19], mouse anti-Wit (1∶500, 23C7, Developmental Studies Hybridoma Bank).

All lysates were diluted in SDS sample buffer to a 1× final concentration, run on a 10% SDS-PAGE gel, transferred to nitrocellulose, and immunoblotting using standard protocols. The HRP signal was detected with ECL Plus (GE Healthcare) and imaged on a Chemi Doc XRS+ Imaging System (BioRad).

### Density Gradient Fractionation

The fractionation protocol was adapted from Phillips et al. [34]. Briefly, ∼0.1 g of heads were collected from 3–5 day old adult Canton S flies. The heads were homogenized in 500 µl of Buffer A (150 mM NaCl; 10 mM Hepes, pH 7.4; 1 mM EGTA; 0.1 mM MgCl_2_) using 20 strokes in a glass-glass homogenizer. The lysate was rotated end over end for 20 min at 4°C and centrifuged at 1000× g for 10 min at 4°C. 150 µl of the resulting supernatant was layered over a 5–25% glycerol gradient on a 50% sucrose pad, and centrifuged at 50,000 rpm for 30 min at 4°C in a TLS-55 rotor (Beckman Coulter). Fifteen fractions (∼133 µl/fraction) were collected from the bottom, diluted in SDS-sample buffer, and separated by SDS-PAGE. Following transfer to nitrocellulose, proteins were detected using antibodies as indicated in the figure.

### Immunohistochemistry

3^rd^ Instar Larvae were dissected in Ca^2+^-free saline (HL-3 pH 7.2, [31]) and fixed in 4% formaldehyde in HL3 or 4% PKS [35] for 15 minutes followed by 2×10 minute washes in 1× PBS (pH 7.2). Primary antibodies were incubated overnight at 4°C in 1xPBX (1xPBS containing 0.1% Triton-X) using the following dilutions: anti-pMad (PS1,1∶500, [36]); anti-Rab11, 1∶100 (BD Biosciences); anti-Rab5, 1∶50 (Abcam); anti-Lqf, 1∶1000 [19]; Alexafluor 488 or AMCA conjugated anti-HRP, 1∶100 (Jackson Immunoresearch); anti-CSP, 1∶50 (ab49, Developmental Studies Hybridoma Bank, DSHB); and anti-GFP, 1∶1000 (Invitrogen). Secondary antibodies conjugated to either Alexafluor 488, Rhodamine-Red X, or Dylight 594 (Jackson Immunoresearch) were used at a concentration of 1∶100. Fixed specimens were mounted in Vectashield (Vector Laboratories) and imaged using an LSM Olympus FV1000 Microscope. Images were taken using a Plan-Apochromat 60× (1.42 NA) and analyzed using ImageJ (NIH).

## Results

### Lqf interacts genetically and biochemically with the BMP receptor Wishful thinking (Wit)

**Figure 1:**
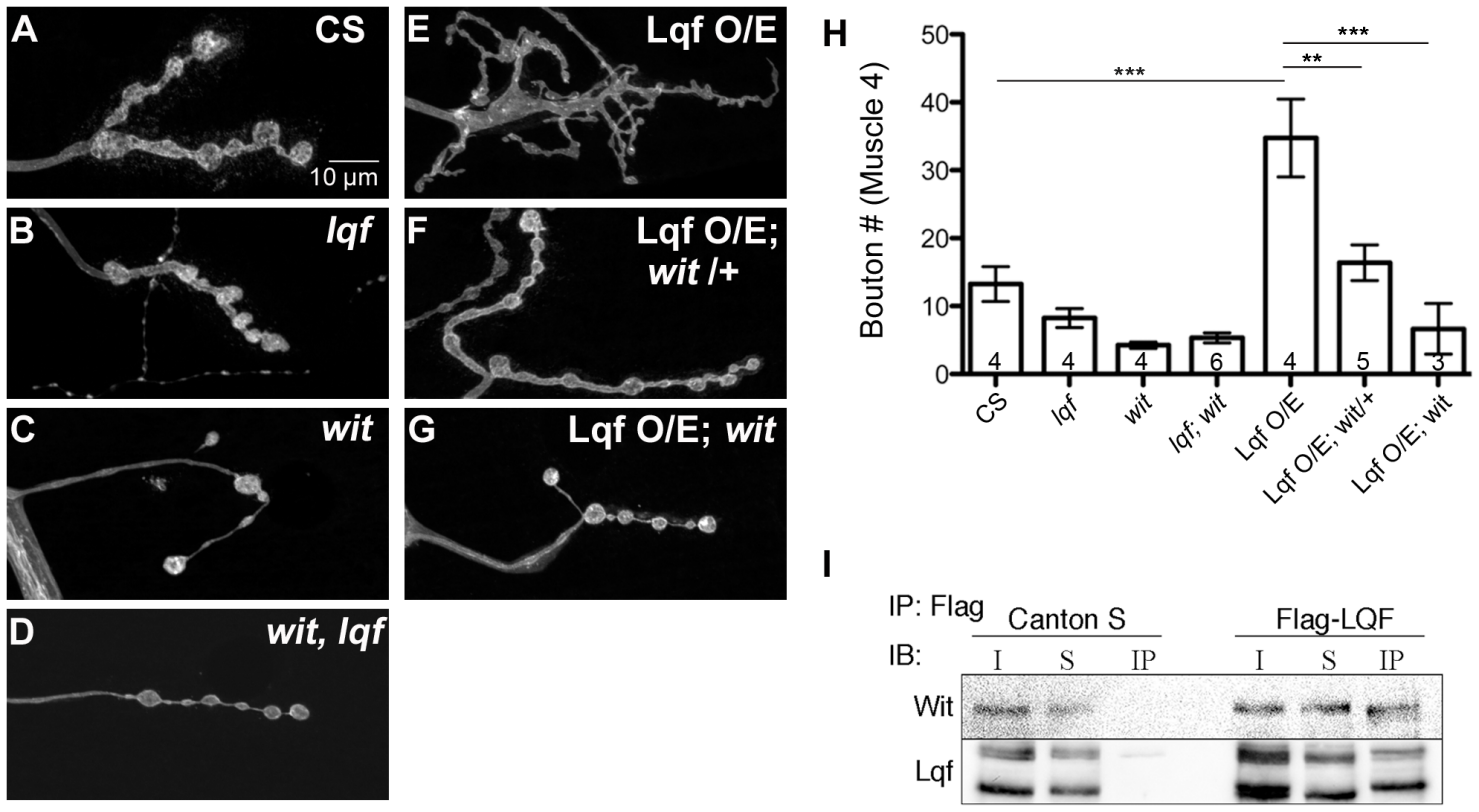
Lqf interacts with Wishful thinking (Wit) to regulate synapse growth. (A–G) Representative images of synaptic bouton morphology in *Drosophila* 3^rd^ instar larval NMJs from the indicated genotypes. Compared to control boutons (A), both *lqf* (*lqf^ARI^*/*lqf* ^FDD9^) and *wit* mutants (*wit* ^B11^/*wit* ^A12^) (B and C, respectively), have fewer boutons, as does the *wit, lqf* double mutant (*wit^A12^*/*wit^B11^*; *lqf^ARI^*/*lqf* Df; D). Neuronal overexpression of Lqf induces synaptic growth with increased number of branches and small satellite boutons and the presence of abnormally large ‘growth cone’-like boutons (Elav^C155^-Gal4/+; UAS-Lqf/+; E), which is suppressed partially by removal of a single copy of *wit* (Elav^C155^-Gal4/+; UAS-Lqf/+; *wit^A12^/+*; F), or suppressed completely by removal of both copies of *wit* (Elav^C155^-Gal4/+; UAS-Lqf/+; *wit^A12^/wit^B11^*; G). H). Quantification of A–G. Error bars represent SEM, n values represent the number of animals per genotype. *P<0.05, **P<0.01, ***P<0.001. One-way ANOVA with Tukey's Multiple Comparison Post test. (I) Co-immunoprecipitation of Flag-tagged Lqf from adult brain lysates from either control flies (containing no Flag), or flies expressing Flag-tagged Lqf, shows Wit co-immunoprecipitates with Lqf. Abbreviations are as follows: Immunoprecipitation (IP); Immunoblot (IB); Input (I); Supernatant (S); Immunoprecipitate (IP).

*Drosophila* 3^rd^ instar larval NMJ boutons display a stereotypic growth pattern, with most significant expansion occurring from 2^nd^ instar to 3^rd^ instar to match the enlargement of body wall muscles [37]. A well understood signaling pathway controlling synaptic growth is the BMP pathway. Mutants deficient in BMP signaling have fewer boutons [5]–[8], [11], [14], whereas enhancement of BMP signaling increases bouton number [29]. Since changes in neuronal Lqf levels also alter bouton number [24], we asked whether Lqf regulated BMP signaling at the larval NMJ by performing genetic epistasis experiments between *lqf* and the BMP receptor mutant, *wit*. Consistent with previous reports, both an *lqf* hypomorph (*lqf^ARI^/lqf^FDD9^*) and a *wit* null mutant *(wit^A12^/wit^B11^)* have a reduction in bouton number relative to control (Fig. 1B, C and H). Genetically elevating neuronal levels of Lqf induces a substantial increase in the number of synaptic protrusions or miniature-synaptic boutons at the NMJ (Elav^C155^-Gal4/+; UAS-Lqf/+; Fig. 1E and H). This overexpression phenotype depends on BMP signaling because removal of a single copy of *wit* results in a significant reduction in bouton number relative to Lqf overexpression (Elav^C155^-Gal4/+; UAS-Lqf/+; *wit^A12^/+*; Fig. 1F and H). Further, neuronal overexpression of Lqf has no effect on synapse growth when the Wit receptor is removed in *wit* mutant backgrounds (Elav^C155^-Gal4/+; UAS-Lqf/+; *wit^A12^*/*wit^B11^*, Fig. 1G and H).

Lqf and its mammalian homolog Epsin1 are known to regulate receptors [21], [38], [39], although an interaction with BMP receptors has not been previously reported. The genetic interaction between *lqf* and *wit* prompted us to next ask whether Lqf physically interacts with Wit. Our co-immunoprecipitation study shows that Wit is present in Lqf immunoprecipitates, but not in the control (Fig. 1I). Together, our genetic and biochemical results suggest that Lqf plays a role in BMP signaling.

### Lqf negatively regulates phosphorylated Mad (pMad) levels at the synapse in a manner similar to other endocytic proteins

**Figure 2:**
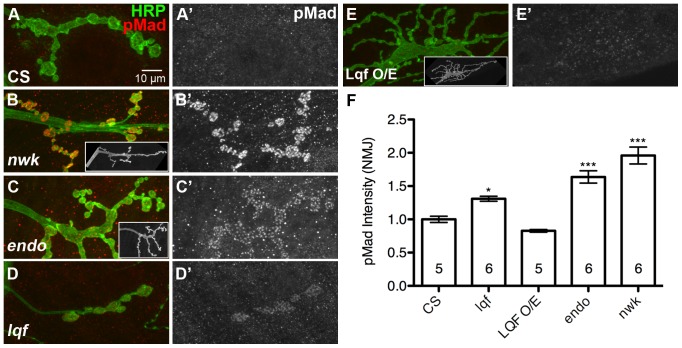
Lqf and other endocytotic proteins negatively regulate pMad at the larval NMJ. (A–E') Representative images of pMad (red) in *Drosophila* 3^rd^ instar larval NMJs. Neuronal membranes are marked by HRP (green). (A'–E') show pMad signal alone. In (B and C) apparent synaptic overgrowth corresponds to increases in synaptic pMad levels for *nwk* and *endo* mutants compared to that in the wild type control (CS, A). (D and F') Synaptic levels of pMad are also significantly elevated in *lqf* mutants, but there is no corresponding NMJ overgrowth. Neuronal overexpression of Lqf (Elav^C155^-Gal4/+; UAS-Lqf/+) causes both a reduction in synaptic pMad levels, and dramatic overgrowth (E and F). (F) Quantification of pMad signal in A–E. Error bars represent SEM, three animals per genotype were quantified, n values represent the number of NMJs for each genotype. *P<0.05, **P<0.01, ***P<0.001. One-way ANOVA with Tukey's Multiple Comparison Post test. Control (CS), *nwk* (*nwk^1^*/*nwk^1^*), *endo* (*endo^A^*/*endo^Δ4^*), *lqf* (*lqf^ARI^/lqf^FDD9^*), Lqf^O/E^ (Elav^C155^-Gal4/+; UAS-Lqf/+).

Although it does not play a role in SV recycling [24], Lqf is known to regulate Delta-Notch trafficking and signaling [18], [21], [40]. To better understand how Lqf regulates BMP signaling, we compared the effect of *lqf* deletion on synaptic pMad levels to the deletion of two endocytotic genes known to affect pMad signaling, *endophilin* (*endo^A^/endo* ^Δ4^) [26] and *nervous wreck* (*nwk, nwk^1^*) [41]. Our data support a previous report [29] showing a significant increase in synaptic pMad levels in *nwk* and *endo* mutants (Fig. 2B, C, and F). Similarly, we show that *lqf* mutant (*lqf^ARI^/lqf^FDD9^*) NMJ boutons also have increased levels of pMad compared to control animals (Fig. 2D and F). Conversely, neuronal overexpression of Lqf results in a reduction of pMad fluorescence intensity within synaptic boutons (Elav^C155^-Gal4/+; UAS-Lqf/+; Fig. 2E and F). These data are consistent with a model in which Lqf and endocytotic proteins are negative regulators of BMP signaling.

### Lqf is necessary for maintaining BMP signals inside motoneuron nuclei

Previous studies have established that increased pMad levels correlate directly with synaptic growth. Notably, *endo* and *nwk* mutants display the highest levels of pMad at the NMJ and the most dramatic synaptic overgrowth [29], [42]. An interesting puzzle is that although pMad signal is also increased at the NMJ in *lqf* mutants, synapses fail to overgrow or display satellite boutons in *lqf* mutants (this study; [24]). BMP signaling regulates synaptic growth primarily through retrograde signaling to the nucleus where it regulates transcription of pro-growth signals [7], [11], [12], [43]. Therefore, a possible explanation for the apparent discrepancy between *lqf* mutants and other endocytic mutants may lie in the pMad levels in motoneuron nuclei.

**Figure 3:**
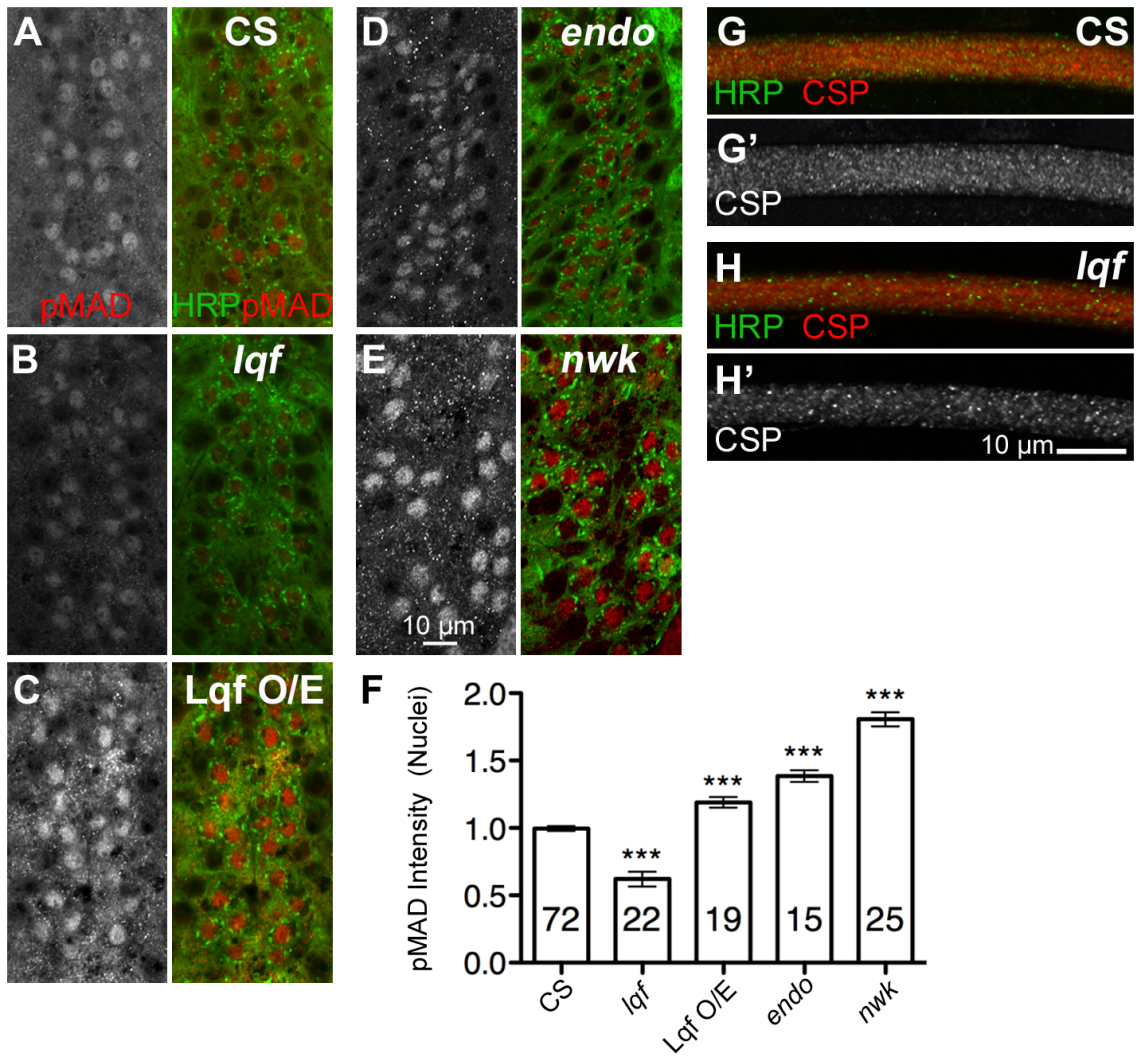
pMad fails to accumulate in the nucleus of motoneurons in *lqf* mutants. (A–E) Representative images of motoneuron nuclei in *Drosophila* 3^rd^ instar larval ventral nerve cord (VNC). pMad signal alone is shown on the left, whereas neuronal membrane (marked by HRP, green) and pMad (red) are shown on the right. (B) In *lqf* mutants, nuclear pMad is significantly reduced relative to control (CS, A), whereas neuronal overexpression of Lqf results in an increase in nuclear localized pMad (C). Similarly, two other endocytotic mutants *endo* (D) and *nwk* (E) have increased levels of nuclear pMad. (F) Quantification of nuclear pMad levels in A–E. Error bars represent SEM. *P<0.05, **P<0.01, ***P<0.001. One-way ANOVA with Tukey's Multiple Comparison Post test. (G–H') Dissected and fixed 3^rd^ Instar larvae were stained with anti-CSP, and segmental nerves (which contain hundreds axons of sensory and motoneurons) were examined for general traffic defects, which would be apparent as CSP accumulations. There are no significant CSP accumulations in either the wild type or *lqf* mutants. Scale bars are 10 µm in all images. n values denote number of nuclei for each genotype, quantified from three animals per genotype. Control (CS), *nwk* (*nwk^1^/nwk^1^*), *endo* (*endo^A^/endo^Δ4^*), *lqf* (*lqf^ARI^/lqf^FDD9^*), Lqf^O/E^ (Elav^C155^-Gal4/+; UAS-Lqf/+).

To address this question, we quantified pMad intensity in individual motoneuron nuclei and showed a significant reduction of nuclear pMad signals in *lqf* mutants (Fig. 3 B and F). In contrast, neuronal overexpression of Lqf increases nuclear pMad levels (Fig. 3C and F). We also found that pMad accumulated significantly more in motoneuron nuclei of *endo* and *nwk* mutants compared to wildtype controls (Fig. 3D–F).

To determine if the decreased levels of nuclear pMad in *lqf* mutant motoneurons are due to a general defect in axonal retrograde trafficking, we examined 3^rd^ instar larval segmental nerves for accumulations of the SV protein cystein-string protein (CSP, [44]). This is a commonly used assay where retrograde axonal trafficking defects are reflected by significant accumulations, termed ‘traffic jams’, of SV proteins [45], [46]. Our data show that there is no discernable difference in CSP accumulation or the presence of traffic jams along the segmental nerves between the controls and *lqf* mutants (Fig. 3 G and H). The absence of axonal traffic jams was also observed using a second SV marker, Synaptotagmin I (Syt I, data not shown; [47], [48]). These observations suggest that Lqf plays a specific and positive role in pMad accumulation in motoneuron nuclei and lend further support to the notion that nuclear pMad is critical for synaptic growth.

### Lqf is required for both nuclear accumulation of pMad and synaptic overgrowth in spinster mutants with impaired lysosomal degradation

**Figure 4:**
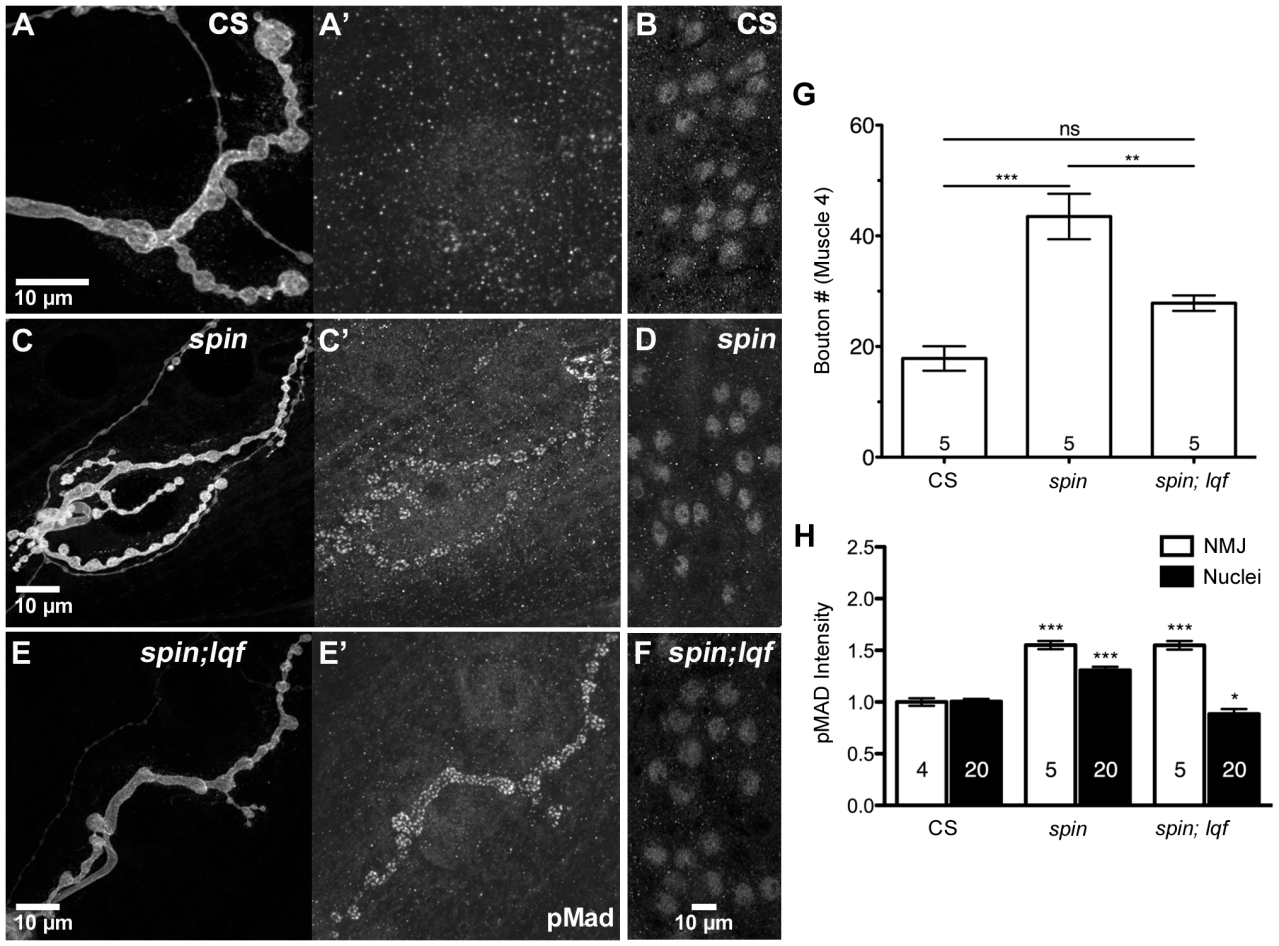
Lqf is required for synaptic overgrowth and pMad retrograde transport in *spinster* mutants. (A–F) Representative images of bouton morphology and pMad levels at the NMJ (A, C, E) and motoneuron nuclei (B, D, F) in *Drosophila* 3^rd^ instar larval NMJs from control larvae (CS, A–B), *spin* mutants (C–D) and *spin;lqf* double mutants (E–F). Synaptic boutons are overgrown in *spin* mutants (C), and this overgrowth is suppressed in the *spin;lqf* double mutants (E). (G) Quantification of synaptic bouton number in *spin* and *spin;lqf* mutants. Taken from three different animals for each genotype, n values represent the number of NMJs quantified. (H) Quantification of pMad intensity in boutons (white bars) and motoneuron nuclei (black bars). n = 5 NMJs from three larvae and 20 nuclei from five different larvae. Error bars represent SEM. *P<0.05, **P<0.01, ***P<0.001. One-way ANOVA with Tukey's Multiple Comparison Post test. Control (CS), *spin* (*spin^4^/spin^5^*), *spin, lqf* (*spin^4^/spin^5^; lqf^ARI^/lqf Df*).

There are multiple routes a cargo can take through the endocytic pathway following internalization. One of the best-characterized routes is trafficking to the lysosome, in which the cargo is first internalized by clathrin-mediated endocytosis and delivered to early endosomes. Endosomes containing cargo destined for lysosomes mature to become late endosomes/MVBs, which can fuse with lysosomes where the cargo is degraded [49]. In flies, the product of the *spinster* (*spin*) gene is shown to be involved in trafficking along the lysosomal degradation pathway [27]. Flies lacking *spin* display BMP signaling-dependent synaptic overgrowth [27]. We first examined bouton morphology in *spin* mutants (*spin^4^/spin^5^*) and in *spin* and *lqf* double mutants (*spin^4^/spin^5^; lqf^ARI^/lqf Df*). *spin* mutants display overgrowth and hyperbranching of NMJs (Fig. 4A and B), similar to those publishes previously [27]. The *spin; lqf* double mutants show reduced synaptic growth similar to those seen in *lqf* (*lqf^ARI^/lqf^FDD9^*) mutants (Fig. 4E and G), suggesting that overgrowth in *spin* mutants depends on Lqf.

Whether the synaptic overgrowth in *spin* mutants correlates with an increase in NMJ or nuclear pMad levels has not been examined. Here we show that BMP signaling is upregulated at both NMJs and motoneuron nuclei in *spin* mutants (Fig. 4C, D, H). This upregulation of pMad signals likely results from a failure to deliver pMad to lysosomes for degradation [27]. We then asked whether there would be a corresponding reduction in nuclear pMad levels along with the suppression of NMJ overgrowth in the *spin; lqf* double mutant. Indeed, the nuclear pMad levels were reduced in *spin; lqf* double mutants (Fig. 4F, H) compared to *spin* mutants and the wildtype larvae (Fig. 4A, H). These results suggest that Lqf functions upstream of Spin in regulating pMad signaling and further demonstrate that failure of nuclear accumulation of pMad in the absence of Lqf account for the lack of synaptic overgrowth in the *spin; lqf* double mutant.

### Lqf regulates endosomal maturation

Our observations thus far show that Lqf is unique amongst endocytic proteins in that it positively regulates synaptic growth while other endocytic proteins constrain synaptic growth. Moreover, pMad fails to accumulate inside the motor nuclei in the absence of Lqf. If *lqf* mutations do not affect axonal transport generally, then how might it affect pMad levels in motoneuron nuclei? Potential mechanisms to explain the *lqf* mutant phenotype include defective processing of the signaling complex through the endocytic pathway (such as degradation of pMad or mis-sorting of BMP signaling endosomes into proper cargo motors) or failure to enter the nucleus. We have examined pMad signals in motoneuron cytoplasm and failed to observe accumulation of pMad outside the nuclei. Hence, we focus on endocytotic processing of BMP signaling endosomes.

We next examined the localization of Lqf and showed that Lqf migrates primarily in low-density fractions of the gradient and in vesicle fractions positive for Rab11 and Rab5 (Fig. S1). Surprisingly, only low levels of Lqf overlap with the plasma membrane marker, Syntaxin 1A, suggesting that Lqf interaction with the plasma membrane is minimal or transient. To ensure that different vesicle populations were migrating differently over the gradient, we also probed for the SV marker, Syt I. Indeed, SVs migrate independently of endosomal populations. Immunostaining shows a transient colocalization with Rab11, but not Rab5 (Fig. S1). These data indicate that Lqf is localized to endosomal vesicles and therefore may have novel functions downstream of endocytosis in nerve cells.

**Figure 5:**
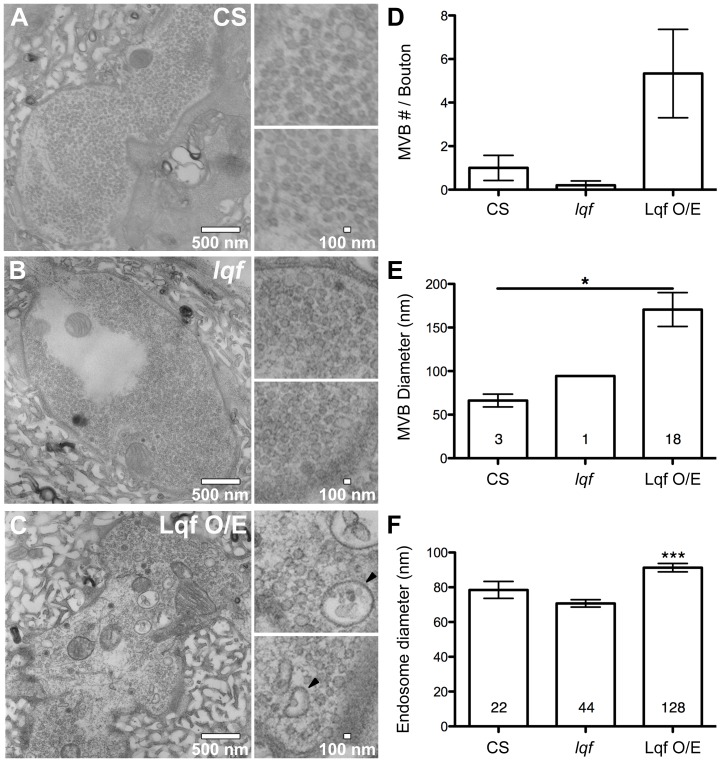
Lqf positively regulates multivesicular body (MVB) formation. (A–C) Ultrastructure of 3^rd^ instar NMJs from the indicated genotypes. Control (A) and *lqf* mutant (B) boutons display a typical vesicle size and distribution. Overexpression of Lqf (C) results in an increased vesicle size and more MVBs that are also increased in size compared to control synapses. Lqf levels do not affect non-multivesicular endosome diameter. Scale bars are 500 µm. (D–F) Quantification of multivesicular bodies (MVBs) and endosomes in A–C. MVB number (D), diameter (E) and endosome diameter (F) have significant differences among control and *lqf* mutants and Lqf overexpression. Error bars represent SEM. *P<0.05, ***P<0.001. (E) Unpaired t-test, *p* = 0.0470. (F) One-way ANOVA, *p* = <0.001. Control (CS), *lqf* (*lqf^ARI^/lqf^FDD9^*), Lqf^O/E^ (Elav^C155^-Gal4/+; UAS-Lqf/+).

We next examined the ultrastructure of endosomes at NMJs of larva either deficient of Lqf (Fig. 5B) or overexpressing Lqf (Fig. 5C). We first quantified the number and size of MVBs, a population of endosomes that form downstream of early endosomes (reviewed in [49], [50]). Whereas the MVB subset of endosomes can be observed, albeit infrequently, in control NMJs, the presence of MVBs at NMJs in the *lqf* mutant is rare (Fig. 5B,D), a finding consistent with a recent report in *lqf* mutant fat cells [51]. Conversely, overexpression of Lqf leads to a dramatic increase in both the number and size of MVBs in NMJs (Fig. 5C, D, E). We then quantified the diameter of non-multivesicular endosomes, and detected a slight, but non-significant decrease in *lqf* mutant NMJs (Fig. 5F). We also detected an increase in endosome size in larvae with Lqf overexpression compared to control NMJs (Fig. 5F). Together, our data along with those of Csikos and colleagues [51] suggest a functional role for Lqf in late stages of endocytic trafficking and MVB formation.

## Discussion

**Figure 6:**
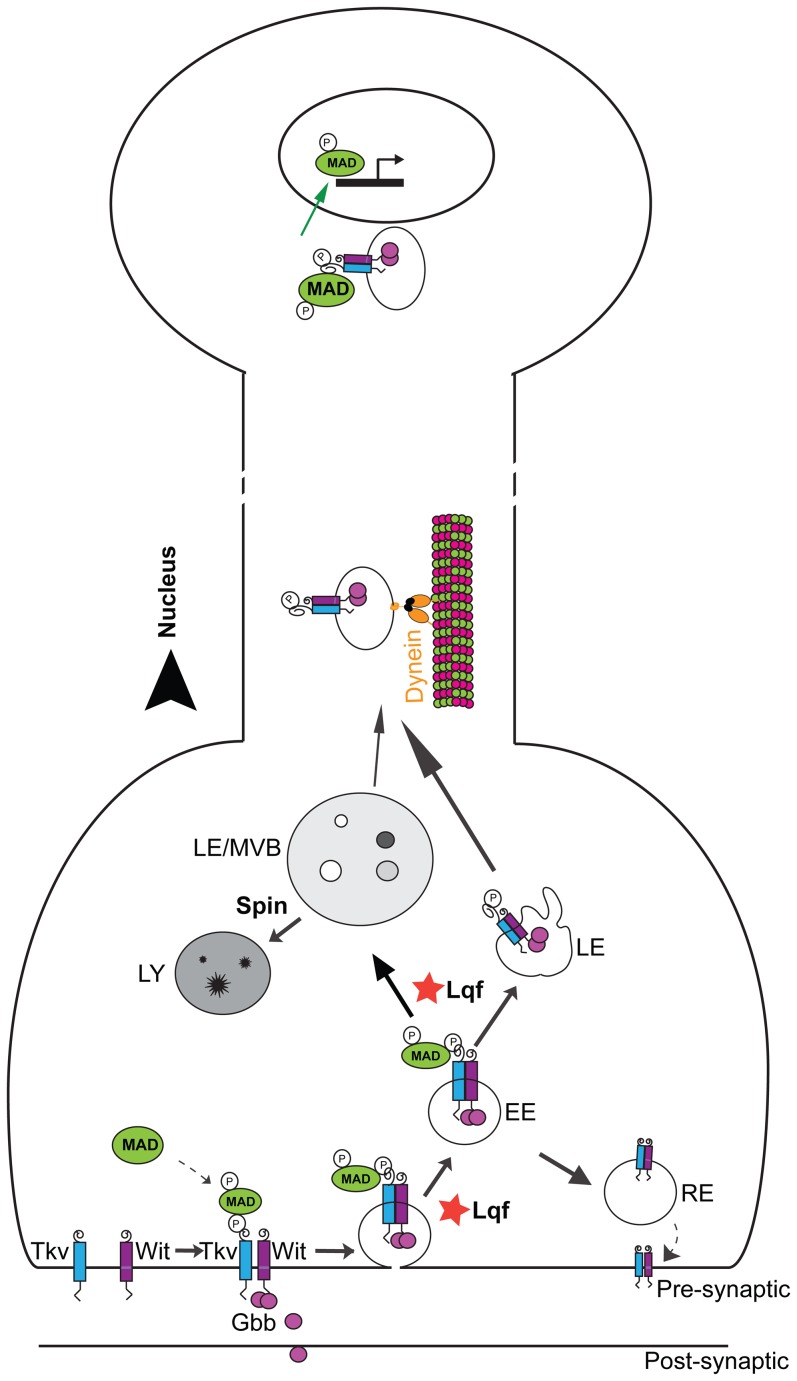
Lqf regulates pMad levels in motoneuron nuclei. Our experimental data support a working model in which Lqf negatively regulates pMad locally at the NMJ but promotes BMP signaling endosome trafficking to the nucleus. At the plasma membrane, Lqf may attenuate receptor activation and consequent phosphorylation of the BMP effector, Mad by promoting internalization of BMP receptors. Following endocytosis, the BMP signaling complex can traffic along multiple routes, including trafficking to the lysosome for degradation (Spin-dependent), recycling to the plasma membrane, or trafficking in a dynein-dependent manner to the nucleus. Our data suggest that Lqf positively regulates movement of pMad to late endosomes/MVBs and shuttles the BMP signaling endosome to an endosomal population destined for the nucleus. EE, early endosome; RE, recycling endosome; LE, late endosome; MVBs, multivesicular body; LY, lysosome.

There is a growing interest in understanding the mechanisms by which signaling molecules are processed and sorted intracellularly. In the current study, we show that Lqf may play a negative role in BMP signaling at the plasma membrane at the NMJ but a positive role in pMad nuclear accumulation (Fig. 6). The increase in local (NMJ) pMad levels in the *lqf* mutant may be explained by a failure to internalize the BMP receptor from the plasma membrane, a hyperactivated signaling endosome, a slowed degradation of pMad or an inability to transport BMP signaling endosome retrogradely into motoneuron nuclei. Our observations on the apparent absence of axonal traffic jams along the segmental nerve are not consistent with a significant axonal transport defect, but we cannot rule out minor changes in retrograde transport rate. While the precise mechanisms remain to be further elucidated, our data suggest a role for Lqf in endosomal function evidenced by the endosomal presence of Lqf and positive regulation of the formation of large MVBs by Lqf.

Wit is proposed to phosphorylate Mad in early endosomes following receptor internalization [52]. Following Mad phosphorylation, the signaling complex may either traffic to the lysosome or be delivered to the nucleus (Fig. 6). We show an increased pMad presence in motoneuron nuclei of *spin* mutants and that *lqf* mutant suppression of synaptic overgrowth in *spin* mutants coincides with a reduction in pMad signal in the motoneuron nuclei. As we do not detect major defects in general axonal transport in *lqf* mutants, we postulate that Lqf either regulates the maturation of BMP signaling endosomes or sorts these endosomes to the appropriate cargo motors prior to retrograde transport.

As shown in *nwk*, *endo*, *ema*, and *dCip4* mutants, there is a positive correlation between NMJ pMad levels and synaptic growth [8], [12], [13], [41]. Our study shows for the first time that pMad levels are increased at both NMJs and motoneuron nuclei in *nwk*, *endo*, and *spin* mutants with synaptic overgrowth. A recent study of the glia-secreted TGFβ ligand Maverick also demonstrates a positive correlation of pMad levels at both the NMJ and motoneuron nuclei with synaptic growth [53]. These observations are consistent with previous studies showing that NMJ fails to grow properly when pMad levels are reduced at both NMJs and motoneuron nuclei in *drich* mutants [12] and *trio* mutants [7]. These studies highlight the importance of nuclear pMad, which alters genes transcription to regulate synaptic growth [7]. In the case of the *spin* mutant, additional mechanisms such as increased oxidative stress and activation of a JNK signaling pathway may co-exist [54]. However, it appears that pMad signaling is rather complex, as synaptic growth and function are impaired in *importin-β11* mutants in which pMad levels are reduced at the NMJ but unaffected in motoneuron nuclei [55]. It remains to be determined how local (NMJ) pMad is regulated and what it does in the synaptic development and function. Similar to what we have observed in *lqf* mutants and *spin; lqf* double mutants, the *nemo* kinase mutants show NMJ growth defects and also have high pMad levels at the NMJ and reduced pMad levels in motoneuron nuclei [43]. Hence, these observations support the notion that there are two separate pools of pMad, NMJ and nuclei [9], and that pMad is needed both locally at the NMJ and globally at the nucleus to stimulate synaptic growth. It appears that both Lqf and Nemo kinase regulate the turnover of pMad at the NMJ and direct BMP signaling endosomes to the motoneuronal nucleus.

The TGFβ/BMP signaling pathways are highly conserved among animals but regulate a large number of diverse processes, including axon guidance [56], neuroprotection after nerve injury [57], dendritic development [58], [59], formation of whisker somatosensory maps [60], and behaviors [61]–[65]. More relevant to the current study, TGFβ signaling also plays important roles in synaptic development, function and plasticity in different animals [66]–[69]. During trigeminal nerve development, target-derived growth factors such as BDNF (brain-derived neurotrophic factor) [70] and BMP4 [71] interplay to regulate BMP retrograde signaling. The BMP effector Smad (equivalent to pMad in flies) is synthesized locally in axons, which requires local signaling of BDNF [72]. It is interesting to note that signaling endosomes are commonly required for both nerve growth factor and BMP signaling and conserved from flies to mammals ([9], [73]; and this study).

Genetic studies of BMP signaling have shed important insights into various neurological disorders (reviewed by [15], [74]). In our study we demonstrate the involvement of BMP retrograde signaling in fly models of lysosomal storage disease (*spin*). The mutant phenotypes of *spin*, including the upregulation of BMP signaling, are similar to those found in the *ema* mutant. The *ema* gene codes for Endosomal maturation defective (Ema), a homolog of the human *Clec16A* gene linked to multiple sclerosis [15], [75]. Our study suggests that Lqf is involved in endosomal maturation to ensure that pMad signaling is relayed to motor nuclei. The findings reported here may advance the understanding of cellular and molecular mechanisms of endosome-related neurological disorders.

## Supporting Information

Figure S1 **Biochemical and immunocytochemical analysis of Lqf subcellular localization.** (A) Total brain lysate was isolated from adult, wild type flies and separated over a 5–25% glycerol gradient. A total of 15 fractions were collected from the top, diluted in SDS-sample buffer, separated over an SDS-PAGE gel, transferred to nitrocellulose, and probed with the indicated antibodies. Lqf migrates to the same fractions as Rab5 and Rab11, at low levels with the plasma membrane fraction (marked by Syntaxin 1A, Syx) and with Synaptotagmin I (Syt I)-positive synaptic vesicle pools. (B and C) Representative images of wild-type *Drosophila* 3^rd^ instar larvae stained with Lqf (red) and either Rab5 (B, green), or Rab11 (C, green). Lqf- and Rab11-positive punctae are found to colocalize in a small subset of vesicles (C, arrowheads).
(TIF)
