# Supplementary figures and images for: Epsin 1 Promotes Synaptic Growth by Enhancing BMP Signal Levels in Motoneuron Nuclei
Source: PLoS One. 2013 Jun 19;8(6):e65997. doi: 10.1371/journal.pone.0065997 (PMC3686817; doi:10.1371/journal.pone.0065997)

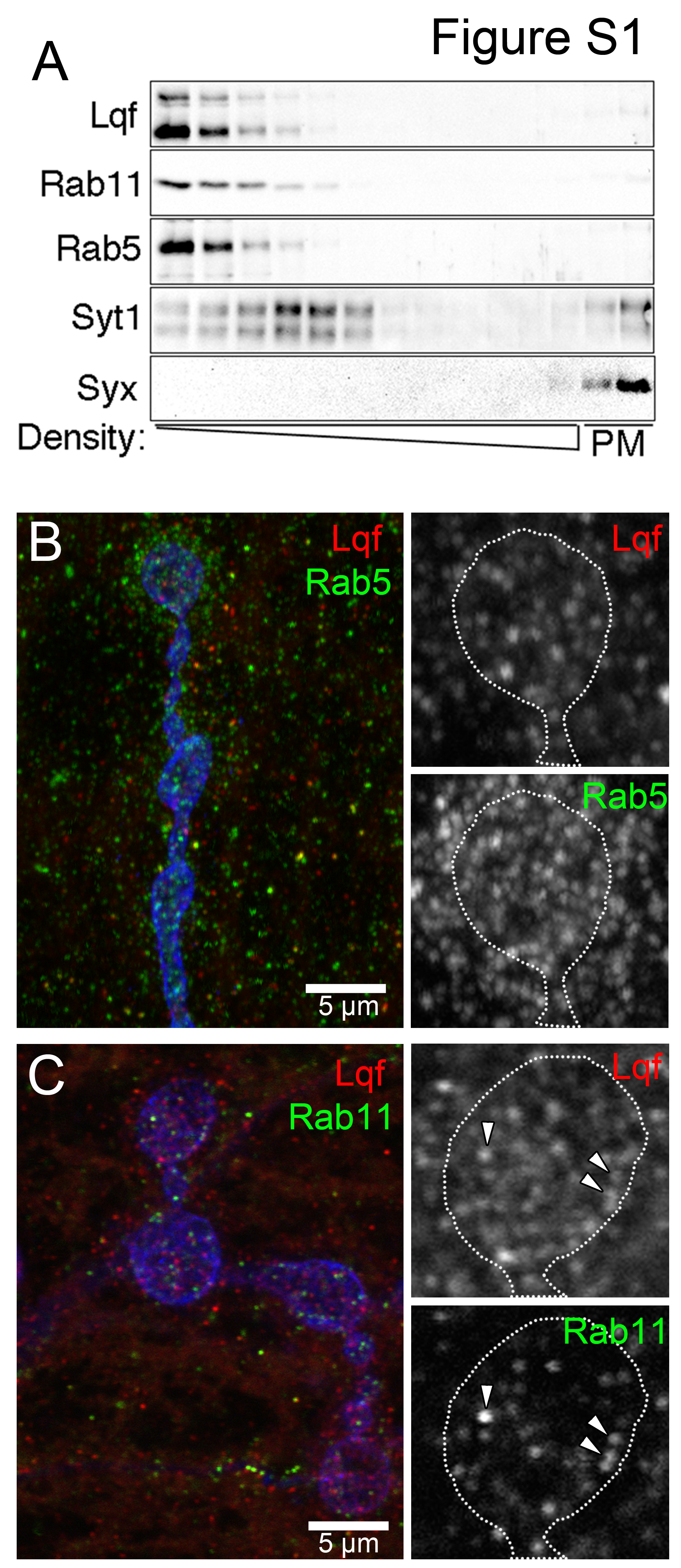

Supplement: Figure S1 — Biochemical and immunocytochemical analysis of Lqf subcellular localization. (A) Total brain lysate was isolated from adult, wild type flies and separated over a 5–25% glycerol gradient. A total of 15 fractions were collected from the top, diluted in SDS-sample buffer, separated over an SDS-PAGE gel, transferred to nitrocellulose, and probed with the indicated antibodies. Lqf migrates to the same fractions as Rab5 and Rab11, at low levels with the plasma membrane fraction (marked by Syntaxin 1A, Syx) and with Synaptotagmin I (Syt I)-positive synaptic vesicle pools. (B and C) Representative images of wild-type Drosophila 3rd instar larvae stained with Lqf (red) and either Rab5 (B, green), or Rab11 (C, green). Lqf- and Rab11-positive punctae are found to colocalize in a small subset of vesicles (C, arrowheads). (TIF) [file pone.0065997.s001.tif]
